# Supplementary material for: Heat adaptation of phage T7 under an extended genetic code
Source: Virus Evol. 2021 Dec 1;7(2):veab100. doi: 10.1093/ve/veab100 (PMC8923235; doi:10.1093/ve/veab100)
Supplement: veab100_Supp [file veab100_supp.zip › Sequencing_Analysis.zip!/Sequencing_Analysis/S2/evidence/JC_SIDE_1_67.html]

BRESEQ :: Evidence


| New junction evidence | | | | | | | | | | | |
| --- | --- | --- | --- | --- | --- | --- | --- | --- | --- | --- | --- |
|  | | seq id | position | reads (cov) | reads (cov) | score | skew | freq | annotation | gene | product |
| \* | ? | NC\_001604.1 | = 16508 | 3993 (0.750) | 107 (0.020) | 85/586 | 24.4 | 2.8% | intergenic (–/–) | *–/–* | –/– |
|  | ? | NC\_001604.1 | 16970 = | 3426 (0.660) |  |  |  |  | intergenic (–/–) | *–/–* | –/– |
| Rejected: Coverage evenness skew score above cutoff. | | | | | | | | | | | |
| Rejected: Frequency below/above cutoff threshold. | | | | | | | | | | | |

Only 100 of 5222 total aligned reads displayed.  

`GCTCTCATCTGCAAACTGTGGATTATCAAGACCGAAGAGATGCTCGTAGAGAAAGGCTTGAAGCATGGCTGGGATGGGGACTTTGCGTACATGGCATGGGTACATGATGAAATCCAAGTAGGCTGCCGTACCGAAGAGATTGCTCAGGTGGTCATTGAGACCGCACAAGAAGCGATGCGCTGGGTTGGAGACCACTGGAACTTCCGGTGTCTTCTGGATACCGAAGGTAAGATGGGTCCTAATTGGGCGATTTGCCACTGATACAGGAGGCTACTCATGAACGAAAGACACTTAACAGGTGCTGCTTCTGAAATGCTAGTAGCCTACAAATTTACCAAAGCTGGGTACA‑CTGTCTATTACCCTATGCTGACTCAGAGTAAAGAGGACTTGGTTGTATGTAAGGATGGTAAATTTAGTAAGGTTCAGGTTAAAACAGCCACAACGGTTCAAACCAACACAGGAGATGCCAAGCAGGTTAGGCTAGGTGGATGCGGTAGGTCCGAATATAAGGATGGAGACTTTGACATTCTTGCGGTTGTGGTTGACGAAGATGTGCTTATTTTCACATGGGACGAAGTAAAAGGTAAGACATCCATGTGTGT  >  NC_001604.1/16207‑16808`  

                                                                                                                                                                                                                                                                                                             `|`                                                                                                                                                                                                                                                                                                               
        `cTGCAAACTGTGGATTATCAAGACCGAAGAGATGCTCGTAGAGAAAGGCTTGAAGCATGGCTGGGATGGGGACTTTGCGTACATGGCATGGGTACATGATGAAATCCAAGTAGGCTGCCGTACCGAAGAGATTGCTCAGGTGGTCATTGAGACCGCACAAGAAGCGATGCGCTGGGTTGGAGACCACTGGAACTTCCGGTGTCTTCTGGATACCGAAGGTAAGATGGGTCCTAATTGGGCGATTTGCCACTGATACAGGAGGCTACTCATGAACGAAAGACACTTAACAGGTGCTGCTTCt                                                                                                                                                                                                                                                                                                        >  1:503466/1‑301 (MQ=255)`  
          `gCAAACTGTGGATTATCAAGACCGAAGAGATGCTCGTAGAGAAAGGCTTGAAGCATGGCTGGGATGGGGACTTTGCGTACATGGCATGGGTACATGATGAAATCCAAGTAGGCTGCCGTACCGAAGAGATTGCTCAGGTGGTCATTGAGACCGCACAAGAAGCGATGCGCTGGGTTGGAGACCACTGGAACTTCCGGTGTCTTCTGGATACCGAAGGTAAGATGGGTCCTAATTGGGCGATTTGCCACTGATACAGGAGGCTACTCATGAACGAAAGACACTTAACAGGTGCTGCTTCTGa                                                                                                                                                                                                                                                                                                      >  1:362466/1‑301 (MQ=255)`  
                 `gtgGATTATCAAGACCGAAGAGATGCTCGTAGAGAAAGGCTTGAAGCATGGCTGGGATGGGGACTTTGCGTACATGGCATGGGTACATGATGAAATCCAAGTAGGCTGCCGTACCGAAGAGATTGCTCAGGTGGTCATTGAGACCGCACAAGAAGCGATGCGCTGGGTTGGAGACCACTGGAACTTCCGGTGTCTTCTGGATACCGAAGGTAAGATGAGTCCTAATTGGGCGATTTGCCACTGATACAGGAGGCTACTCATGAACGAAAGACACTTAACAGGTGCTGCTTCTGAAATGCta                                                                                                                                                                                                                                                                                               >  1:195171/1‑301 (MQ=255)`  
                   `ggATTATCAAGACCGAAGAGATGCTCGTAGAGAAAGGCTTGAAGCATGGCTGGGATGGGGACTTTGCGTACATGGCATGGGTACATGATGAAATCCAAGTAGGCTGCCGTACCGAAGAGATTGCTCAGGTGGTCATTGAGATCGCACAAGAAGCGATGCGCTGGGTTGGAGACCACTGGAACTTCCGGTGTCTTCTGGATACCGAAGGTAAGATGGGTCCTAATTGGGCGATTTGCCACTGATACAGGAGGCTACTCATGAACGAAAGACACTTAACAGGTGCTGCTTCTGAAATGCtagt                                                                                                                                                                                                                                                                                             >  1:648733/1‑301 (MQ=255)`  
                   `ggATTATCAAGACCGAAGAGATGCTCGTAGAGAAAGGCTTGAAGCATGGCTGGGATGGGGACTTTGCGTACATGGCATGGGTACATGATGAAATCCAAGTAGGCTGCCGTACCGAAGAGATTGCTCAGGTGGTCATTGAGACCGCACAAGAAGCGATGCGCTGGGTTGGAGACCACTGGAACTTCCGGTGTCTTCTGGATACCGAAGGTAAGATGGGTCCTAATTGGGCGATTTGCCACTGATACAGGAGGCTACTCATGAACGAAAGACACTTAACAGGTGCTGCTTCTGAAATGCtagt                                                                                                                                                                                                                                                                                             >  1:685252/1‑301 (MQ=255)`  
                   `ggATTATCAAGACCGAAGAGATGCTCGTAGAGAAAGGCTTGAAGCATGGCTGGGATGGGGACTTTGCGTACATGGCATGGGTACATGATGAAATCCAAGTAGGCTGCCGTACCGAAGAGATTGCTCAGGTGGTCATTGAGACCGCACAAGAAGCGATGCGATGGGTTGGAGACCACTGGAACTTCCGGTGTCTTCTGGATACCGAAGGTAAGATGGGTCCTAATTGGGCGAGTTGCCACTGATACAGGAGGCTACTCATGAACGAAAGACACTTAACAGGTGCTGCTTCTGAAATGCtgtc                                                                                                                                                                                                                                                                                             >  1:174444/1‑298 (MQ=255)`  
                     `tgtataagagacagagagATGCTCGTAGAGAAAGGCTTGAAGCATGGCTGGGATGGGGACTTTGCGTACATGGCATGGGTACATGATGAAATCCAAGTAGGCTGCCGTACCGAAGAGATTGCTCAGGTGGTCATTGAGACCGCACAAGAAGCGATGCGCTGGGTTGGAGACCACTGGAACTTCCGGTGTCTTCTGGATACCGAAGGTAAGATGGGTCCTAATTGGGCGATTTGCCACTGATACAGGAGGCTACTCATGAACGAAAGACACTTAACAGGTGCTGCTTCTGAAATGCtagtag                                                                                                                                                                                                                                                                                           <  1:224209/287‑1 (MQ=255)`  
                      `gtATCAAGACCGAAGGGATGCTCGTAGAGAAAGGCTTGAAGCATGGCTGGGATGGGGACTTTGCGTACATGGCATTGGTACATGATGAAATCCAAGTAGGCTGCCGTACCGAAGAGATTGCTCAGGTGGTCATTGAGACCGCACAAGAAGCGATGCGCTGGGTTGGAGACCACTGGAACTTCCGGTGTCTTCTGGATACCGAAGGTAAGATGGGTCCTAATTGGGCGATTTGCCACTGATACAGGAGGCTACTCATGAACGAAAGACACTTAACAGGTGCTGCTTCTGAAATGCTAGTAGc                                                                                                                                                                                                                                                                                          <  1:815963/300‑1 (MQ=255)`  
                        `aTCAAGACCGAAGAGATGCTCGTAGAGAAAGGCTTGAAGCATGGCTGGGATGGGGACTTTGCGTACATGGCATGGGTACATGATGAAATCCAAGTAGGCTGCCGTACCGAAGAGATTGCTCAGGTGGTCATTGAGACCGCACAAGAAGCGATGCGCTGGGTTGGAGACCACCGGAACTTCCGGTGTCTTCTGGATACCGAAGGTAAGATGGGTCCTAATTGGGCGATTTGCCACTGATACAGGAGGCTACTCATGAACGAAAGACACTTAACAGGTGCTGCTTCTGAAATGcctgtctctt                                                                                                                                                                                                                                                                                        >  1:641539/1‑292 (MQ=255)`  
                          `gaaagacGAAGAGATGCTCGTAGAGAAAGGCTTGAAGCATGGCTGGGATGGGGACTTTGCGTACATGGCATGGGTACATGATGAAATCCAAGTAGGCTGCCGTACCGAAGAGATTGCTCAGGTGGTCATTGAGACCGCACAAGAAGCGATGCGCTGGGTTGGAGACCACTGGAACTTCCGGTGTCTTCTGGATACCGAAGGTAAGATGTGTCCTAATTGGGCGATTTGCCACTGATACAGGAGGCTACTCATGAACGAAAGACACTTAACAGGTGCTGCTTCTGAAATGCTAGTAGCCTAc                                                                                                                                                                                                                                                                                      <  1:355743/295‑1 (MQ=255)`  
                            `aGACCGAAGAGATGCTCGTAGAGAAAGGCTTGAAGCATGGCTGGGATGGGGACTTTGCGTACATGGCATGGGTACATGATGAAATCCAAGTAGGCTGCCGTACCGAAGAGATTGCTCAGGTGGTCATTGAGACCGCACAAGAAGCGATGCGCTGGGTTGGAGACCACTGGAACTTCCGGTGTCTTCTGGATACCGAAGGTAAGATGGGTCCTAATTGGGCGATTTGCCACTGATACAGGAGGCTACTCATGAACGAAAGACACTTAACAGGTGCTGCTTCTGAAATGCTAGTAGCCTACaa                                                                                                                                                                                                                                                                                    >  1:472791/1‑301 (MQ=255)`  
                            `aGACCGAAGAGATGCTCGTAGAGAAAGGCTTGAAGCATGGCTGGGATGGGGACTTTGCGTACATGGCATGGGTACATGATGAAATCCAAGTAGGCTGCCGTACCGAAGAGATTGCTCAGGTGGTCATTGAGACCGCACAAGAAGCGATGCGCTGGGTTGGAGACCACTGGAACTTCCGGTGTCTTCTGGATACCGAAGGTAAGATGGGTCCTAATTGGGCGATTTGCCACTGATACAGGAGGCTACTCATGAACGAAAGACACTTAACAGGTGCTGCTTCTGAAATGCTAGTAGCCTACaa                                                                                                                                                                                                                                                                                    >  1:538407/1‑301 (MQ=255)`  
                             `aaCCGAAGAGATGCTCGTAGAGAAAGGCTTGAAGCATGTCTGGGATGGGGACTTTGCGTACATGGCATGGGTACATGATGAAATCCAAGTAGGCTGCCGTACCGAAGAGATTGCTCAGGTGGTCATTGAGACCGCACAAGAAGCGATGCGCTGGGTTGGAGACCACTGGAACTTCCGGTGTCTTCTGGATACCGAAGGTAAGATGGGTCCTAATTGGGCGATTTGCCACTGATACAGGAGGCTACTCATGAACGAAAGACACTTAACAGGTGCTGCTTCTGAAATGCTAGTAGCCTACaaa                                                                                                                                                                                                                                                                                   <  1:71511/300‑1 (MQ=255)`  
                              `aataagagacagCTCGTAGAGAAAGGCTTGAAGCATGGCTGGGATGTGGACTTTGCGTACATGGCATGTGTACATGATGAAATCCAAGTAGGCTGCCGTACCGAAGAGATTGCTCAGCTGGTCATTGAGACCGCACAAGAAGCGATGCGCTTGGTTGGATACCACTGGAACTTCCGGTGTCTTCTGGATACCGAAGGTAAGATGGGTCCTAATTGGGCGATTTGCCACTGATACAGGAGGCTACTCATGAACGAAAGACACTTAACAGGTGCTGCTTCTGAAATGCTAGTAGCCTACAAAt                                                                                                                                                                                                                                                                                  <  1:787599/290‑1 (MQ=255)`  
                              `aCCGAAGAGATGCTCGTAGAGAAAGGCTTGAAGCATGGCTGGGATGGGGACTTTGCGTACATGGCATGGGTACATGATGAAATCCAAGTAGGCTGCCGTACCGAAGAGATTGCTCAGGTGGTCATTGAGACCACACAAGAAGCGATGCGCTGGGTTGGAGACCACTGGAACTTCCGGTGTCTTCTGGATACCGAAGGTAAGATGGGTCCTAATTGGGCGATTTGCCACTGATACAGGAGGCTACTCATGAACGAAAGACACTTAACAGGTGCTGCTTCTGAAATGCTAGTAGCCTACAAAt                                                                                                                                                                                                                                                                                  <  1:437311/301‑1 (MQ=255)`  
                                   `agagATGCTCGTAGAGAAAGGCTTGAAGCATGGCTGGGATGGGGACTTTGCGTACATGGCATGGGTACATGATGAAATCCAAGTAGGCTGCCGTACCGAAGAGATTGCTCAGGTGGTCATTGAGACCGCACAAGAAGCGATGCGCTGGGTTGGAGACCACTGGAACTTCCGGTGTCTTCTGGATACCGAAGGTAAGATGGGTCCTAATTGGGCGATTTGCCACTGATACAGGAGGCTACTCATGAACGAAAGACACTTAACAGGTGCTGCTTCTGAAATGCTAGTAGCCTACAAATTTAcc                                                                                                                                                                                                                                                                             <  1:677233/301‑1 (MQ=255)`  
                                   `agagATGCTCGTAGAGAAAGGCTTGAAGCATGGCTGGGATGGGGACTTTGCGTACATGGCATGGGTACATGATGAAATCCAAGTAGGCTGCCGTACCGAAGAGATTGCTCAGGTGGTCATTGAGACCGCACAAGAAGCGATGCGCTGGGTTGGAGACCACTGGAACTTCCGGTGTCTTCTGGATACCGAAGGTAAGATGGGTCCTAATTGGGCGATTTGCCACTGATACAGGAGGCTACTCATGAACGAAAGACACTTAACAGGTGCTGCTTCTGAAATGCTAGTAGCCTACAAATTTAcc                                                                                                                                                                                                                                                                             <  1:599158/301‑1 (MQ=255)`  
                                             `gTAGAGAAAGGCTTGAAGCATGGCTGGGATGGGGACTTTGCGTACATGGCATGGGTACATGATGAAATCCAAGTAGGCTGCCGTACCGAAGAGATTGCTCAGGTGGTCATTGAGACCGCACAAGAAGCGATGCGCTGGGTTGGAGACCACTGGAACTTCCGGTGTCTTCTGGATACCGAAGGTAAGATGGGTCCTAATTGGGCGATTTGCCACTGATACAGGAGGCTACTCATGAACGAAAGACACTTAACAGGTGCTGCTTCTGAAATGCTAGTAGCCTACAAATTTACCAAAGCTGGGt                                                                                                                                                                                                                                                                   <  1:145376/301‑1 (MQ=255)`  
                                               `agagAAAGGCTTGAAGCATGGCAGGGATGGGGACTTTGCGTACATGGCATGGGTACATGATGAAATCCAAGTAGGCTGCCGTACCGAAGAGATTGCTCAGGTGGTCATTGAGACCGCACAAGAAGCGATGCGCTGGGTTGGAGACCACTGGAACTTCCGGTGTCTTCTGGATACCGAAGGTAAGATGGGTCCTAATTGGGCGATTTGCCACTGATACAGGAGGCTACTCATGAACGAAAGACACTTAACAGGTGCTGCTTCTGAAATGCTAGTAGCCTACAAATTTACCAAAGCTGGGTac                                                                                                                                                                                                                                                                 <  1:377845/301‑1 (MQ=255)`  
                                               `agagAAAGGCTTGAAGAATGGCTGGGATGGGGACTTTGCGTACATGGCATGGGTACATGATGAAATCCAAGTAGGCTGCCGTACCGAAGAGATTGCTCAGGTGGTCATTGAGACCGCACAAGAAGCGATGCGCTGGGTTGGAGACCACTGGAACTTCCGGTGTCTTCTGGATACCGAAGGTAAGATGGGTCCTAATTGGGCGATTTGCCACTGATACAGGAGGCTACTCATGAACGAAAGACACTTAACAGGTGCTGCTTCTGAAATGCTAGTAGCCTACAAATTTACCAAAGCTGGGTac                                                                                                                                                                                                                                                                 <  1:692270/301‑1 (MQ=255)`  
                                                 `ggaAAGGCTGGAAGCATGGCTGGGATGGGGACTGTTCGTACATGGCATGGGTACATGATGAAATCCAAGTAGGCTGCCGTACCGAAGAGAGTGCTCAGGTGGTCATTGAGACCGCACAAGAAGCGATGCGCTGGGTTGGAGACCACTGGAACTTCCGGTGTCTTCTGGATACCGAAGGTAAGATGGGTCCTAATTGGGCGATTTGCCACTGATACAGGAGGCTACTCATGAACGAAAGACACTTAACAGGTGCTGCTTCTGAAATGCTAGTAGCCTACAAATTTACCAAAGCTGGGTaca‑c                                                                                                                                                                                                                                                              <  1:447473/300‑1 (MQ=255)`  
                                                 `agaAAGGCTTGAAGCATGGCTGGGAGGGGGACTTTGCGTACATGGCATGGGTACATGATGAAAGCCAAGTAGGCTGCCGTACCGAAGAGATTGCTCAGGTGGTCATTGAGACCGCACAAGAAGCGATGCGCTGGGTTGGAGACCACTGGAACTTCCGGTGTCTTCTGGATACCGAAGGTAAGATGGGTCCTAATTGGGCGATTTGCCACTGATACAGGAGGCTACTCATGAACGAAAGACACTTAACAGGTGCTGCTTCTGAAATGCTAGTAGCCTACAAATTTACCAAAGCTGGGTaca‑c                                                                                                                                                                                                                                                              <  1:565898/301‑1 (MQ=255)`  
                                                     `aGGCTTGAAGCATGGCTGGGATGGGGACTTTGCGTACATGGCATGGGTACATGATGAAATCCAAGTAGGCTGCCGTACCGAAGAGATTGCTCAAGTGGTCATTGAGACCGCACAAGAAGCGATGCGCTGGGTTGGAGACCACTGGAACTTCCGGTGTCTTCTGGATACCGAAGGTAAGATGGGTCCTAATTGGGCGATTTGCCACTGATACAGGAGGCTACTCATGAACGAAAGACACTTAACAGGTGCTGCTTCTGAAATGCTAGTAGCCTACAAATTTACCAAAGCTGGGTACA‑CTGTc                                                                                                                                                                                                                                                          >  1:799761/1‑301 (MQ=255)`  
                                                      `ggCTTGAAGCATGGCTGGGATGGGGACTTTGCGTACATGGCATGGGTACATGATGAAATCCAAGTAGGCTGCCGTACCGAAGAGATTTCTCAGGTGGTCATTGAGACCGCACAAGAAGCGATGCGCTGGGTTGGAGACCACTGGAACTTCCGGTGTCTTCTGGATACCGAAGGTAAGATGGGTCCTAATTGGGCGATTTGCCACTGATACAGGAGGCTACTCATGAACTAAAGACACTTAACAGGTGCTGCTTCTGAAATGCTAGTAGCCTACAAATTTACCAAAGCTGGGTACA‑CTGTCt                                                                                                                                                                                                                                                         >  1:292815/1‑301 (MQ=255)`  
                                                      `ggCTTGAAGCAGGGCTGGGATGGGGACTTTGCGTACATGGCATGGGTACATGATGAAATCCAAGTAGGCTGCCGTACCGAAGAGATTGCTCAGGTGGTCATTGAGACCGCACAAGAAGCGATGCGCTGGGTTGGAGACCACTGGAACTTCCGGTGTCTTCTGGATACCGAAGGTAAGATGGGTCCTAATTGGGAGATTTGCCACTGATACAGGAGGCTACTCATGAACGAAAGACACTTAACAGGTGCTGCTTCTGAAATGCTAGTAGCCTACAAATTTACCAAAGCTGGGTACA‑CTGTCt                                                                                                                                                                                                                                                         <  1:189243/301‑1 (MQ=255)`  
                                                       `gCTTGAAGCATGGCTGGGATGGGGACTTTGCGTACATGGCATGGGTACATGATGAAATCCAAGTAGGCTGCCGTACCGAAGAGATTGCTCAGGTGGTCATTGAGACCGCACAAGAAGCGATGCGCTGGGTTGGAGACCACTGGAACTTCCGGTGTCTTCTTGATACCGAAGGTAAGATGGGTCCTAATTGGGCGATTTGCCACTGATACAGGAGGCTACTCATGAACGAAAGACACTTAACAGGTGCTGCTTCTGAAATGCTAGTAGCCTACAAATTTACCAAAGCTGGGTACA‑CTGTCtt                                                                                                                                                                                                                                                        >  1:72239/1‑300 (MQ=255)`  
                                                           `gAAGCATTGCTGGGATGGTGACTTTGCGTACATGGCATGGGTACATGATGAAATCCAAGTAGGCTTCCGTACCGAAGAGATTGCTCAGGTGGTCATTGAGACCGCACAAGAAGCGATGCGCTGGGTTGGAGACCACTGGAACTTCCGGTGTCTTCTGGATACCTAAGGTAAGATGGGTCCTAATTGGGCGATTTGCCACTGATACAGGAGGCTACTCATGAACGAAAGACACTTAACAGGTGCTGCTTCTGAAATGCTAGTAGCCTACAAATTTACCAAAGCTGGGTACA‑CTGTCTATTAc                                                                                                                                                                                                                                                    <  1:326783/301‑1 (MQ=255)`  
                                                               `cATGGCTGGGATGGGGACTTTGCGTACATGGCATGGGTACATGATGAAATCCAAGTAGGCTGCCGTACCGAAGAGATTGCTCAGGTGGTCATTGAGACCGCACAAGAAGCGATGCGCTGGGTTGGAGACCACTGGAACTTCCGGTGTCTTCTGGATACCGAAGGTAAGATGGGTCCTAATTGGGCGATTTGCCACTGATACAGGAGGCTACTCATGAACGAAAGACACTTAACAGGTGCTGCTTCTGAAATGCTAGTAGCCTACAAATTTACCAAAGCTGGGTACA‑CTGTCTATTACCCTa                                                                                                                                                                                                                                                >  1:510451/1‑301 (MQ=255)`  
                                                                  `ggCTGGGAT‑GGGACTTTGCGTACATGGCATGGGTACATGATGAAATCCAAGTAGGCTGCCGTACCGAAGAGATTGCTCAGGTGGTCATTGAGACCGCACAAGAAGCGATGCGCTGGGTTGGAGACCACCGGAACTTCCGGTGTCTTCTGGATACCGAAGGTAAGATGGGTCCTAATTGGGCGATTTGCCACTGATACAGGAGGCTACTCATGAACGAAAGACACTTAACAGGTGCTGCTTCTGAAATGCTAGTAGCCTACAAATTTACCAAAGCTGGGTACA‑CTGTCTATTAcctgtctct                                                                                                                                                                                                                                            >  1:161912/1‑294 (MQ=255)`  
                                                                       `ggATGGGGACTTTGCGTACATGGCATGGGTACATGATGAAATCCAAGTAGGCTGCCGTACCGAAGAGATTGCTCAGGTGGTCATTGAGACCGCACAAGAAGCGATGCGCTGGGTTGGAGACCACTGGAACTTCCGGTGTCTTCTGGATACCGAAGGTAAGATGGGTCCTAATTGGGCGATTTGCCACTGATACAGGAGGCTACTCATGAACGAAAGACACTTAACAGGTGCTGCTTCTGAAATGCTAGTAGCCTACAAATTTACCAAAGCTGGGTACACCTGTCTCTTatacacatctccg                                                                                                                                                                                                                                         >  1:736273/1‑289 (MQ=255)`  
                                                                        `aaaggggACTTTGCGTACATGGCATGGGTACATGATTAAATCCATGTATGCTGCCGTACCGAAGAGATTACTCAGGTGGTCATTGAGACCGCACAAGAAGCTATGCGCTGGGTTGGAGACCACTGGAAATTCCGGTGTCTGATGGATACCGAAGGTAAGATGGGTCCTAATTGGGCGATTTGAAAATGATACAGGAGGCTAATCATGAACGAAAGACACTTAACAGGAGCTGCTTCTGAAATGCTAGTAGCCTACAAATTTACCAAAGCTGGGTACA‑CTGTCTATTACCCTATGCTGACTc                                                                                                                                                                                                                                       <  1:108016/298‑1 (MQ=255)`  
                                                                            `gggACTTTGCGTACATGGCATGGGTACATGATGAAATCCAAGTAGGCTGCCGTACCGAAGAGATTGCTCAGGTGGTCATTGAGACCGCACAAGAAGCGATGCGCTGGGTTGGAGACCACTGGAACTTCCGGTGTCTTCTGGATACCGAAGGTAAGATGGGTCCTAATTGGGCGATTTGCCACTGATACAGGAGGCTACTCATGAACGAAAGACACTTAATAGGTGCTGCTTCTGAAATGCTAGTAGCCTACAAATTTACCAAAGCTGGGTACA‑CTGTCTATTACCCTATGCTGACTCagag                                                                                                                                                                                                                                   <  1:272021/301‑1 (MQ=255)`  
                                                                                `cTTTGCGTACATGGCATGGGTACATGATGAAATCCAAGTAGGCTGCCGTACCGAAGAGATTGCTCAGGTGGTCATTGAGACCGCACAAGAAGCGATGCGCTGGGTTGGAGACCACTGGAACTTCCGGTGTCTTCTGGATACCGAAGGTAAGATGGGTCCTAATTGGGCGTTTTGCCACTGATACAAGAGGCTACTCATGAACGAAAGACACTTAACAGGTGCTGCTTCTGAAATGCTAGTAGCCTACAAATTTACCAAAGCTGGGTACA‑CTGTCTATTAcccctgtctcttatacacatct                                                                                                                                                                                                                               >  1:91885/1‑282 (MQ=255)`  
                                                                                           `tggcatggGTACATGATGAAATCCAAGTAGGCTGCCGTACCGAAGAGATTGCTCAGGTGGTCATTGAGACCGCACAAGAAGCGATTCGCTGGGTTGGAGACCACTGGAACTTCCAGTGTCTTCTGGATACCGAAGGTAAGATGGGTCCTAATTGGGCGATTTGCCACTGATACAGGAGGCTACTCATGAACGAAAGACACTTAACAGGTGCTGCTTCTGAAATGCTAGTAGCCTACAAATTTACCAAAGCTGGGTACA‑CTGTCTATTACCCTATGCTGACTCAGAGTAAAGAGGACTTGGt                                                                                                                                                                                                                    <  1:24957/301‑1 (MQ=255)`  
                                                                                           `tggcatggGTACATGATGAAATCCAAGTAGGCTGCCGTACCGAAGAGATTGCTCAGGTGGTCATTGAGACCGCACAAGAAGCGATGCGCTGGGTTGGAGACCACTGGAACTTCCGGTGTCTTCTGGATACCGAAGGTAAGATGGGTCCTAATTGGGCGATTTGCCACTGATACAGGAGGCTACTCATGAACGAAAGACACTTAACAGGTGCTGCTTCTGAAATGCTAGTAGCCTACAAATTTACCAAAGCTGGGTACA‑CTGTCTATTACCCTATGCTGACTCAGAGTAAAGAGGACTTGGt                                                                                                                                                                                                                    >  1:34767/1‑301 (MQ=255)`  
                                                                                           `tggcatggGTACATGATGAAATCCAAGTAGGCTGCCGTACCGAAGAGATTGCTCAGGTGGTCATTGAGACCGCACAAGAAGCGATGCGCTGGGTTGGAGACCACTGGAACTTCCGGTGTCTTCTGGATACCGAAGGTAAGATGGGTCCTAATTGGGCGATTTGCCACTGATACAGGAGGCTACTCATGAACGAAAGACACTTAACAGGTGCTGCTTCTGAAATGCTAGTAGCCTACAAATTTACCAAAGCTGGGTACA‑CTGTCTATTACCCTATGCTGACTCAGAGTAAAGAGGACTTGGt                                                                                                                                                                                                                    >  1:488180/1‑301 (MQ=255)`  
                                                                                             `gcatggGTACATGATGAAATCCAAGTAGGCTGCCGTACCGAAGAGATTGCTCAGGTGGTCATTGAGACCGCACAAGAAGCGATGCGCTGGGTTGGAGACCACTGGAACTTCCGGTGTCTTCTGTATACCGAAGGTAAGATGGGTCCTAATTGGGCGATTTGCCACTGATACAGGAGGCTACTCATGAACGAAAGACACTTAACAGGTGCTGCTTCTGAAATGCTAGTAGCCTACAAATTTACCAAAGCTGGGTACA‑CTGTCTATTACCCTATGCTGACTCAGAGTAAagactgtctcttat                                                                                                                                                                                                                  >  1:823839/1‑290 (MQ=255)`  
                                                                                                `tGGGTACATGATGAAATCCAAGTAGGCTGCCGTACCGAAGAGATTGCTCAGGTGGTCATTGAGACCGCACAAGAAGCGATGCGCTGGGTTGGAGACCACTGGAACTTCCGGTGTCTTCTGGATACCGAAGGTAAGATGGGTCCTAATTGGGCGATTTGCCACTGATACAGGAGGCTACTCATGAACGAAAGACACTTAACAGGTGCTGCTTCTGAAATGCTAGTAGCCTACAAATTTACCAAAGCTGGGTACA‑CTGTCTATTACCCTATGCTGACTCAGAGTAAAGAGGACTTGGTTGTAT                                                                                                                                                                                                               >  1:572191‑M1/1‑301 (MQ=255)`  
                                                                                                  `gttacATGATGAAATCCAAGTAGGCTGCCGTACCGAAGAGATTGCTCAGGTGGTCATTGAGACCGCACAAGAAGCGATGCGCTGGGTTGGAGACCACTGGAACTTCCGGCGTCTTCTGGATACCGAAGGTAAGATGGGTCCTAATTGGGCGATTTGCCACTGATACAGGAGGCTACTCATGAACGAAAGACACTTAACAGGTGCTGCTTCTGAAATGCTAGTAGCCTACAAATTTACCAAAGCTGGGTACA‑CTGTCTATTACCCTATGCTGACTCAGAGTAAAGAGGACTTGGTTGTATGg                                                                                                                                                                                                             <  1:670491‑M1/299‑2 (MQ=255)`  
                                                                                                  `ggTACATGATGAAATCCAAGTAGGCTGCCGTACCGAAGAGATTGCTCAGGTGGTCATTGAGACCGCACAAGAAGCGATGCGCTGGGTTGGAGACCACTGGAACTTCCGGTGTCTTCTGTATACCGAAGGTAAGATGGGTCCTAATTGGGCGATTTGCCACTGATACAGGAGGATACTCATGAACGAAAGACACTTAGCAGGTGCTGCTTCTGAAATGCTAGTAGCCTACAAATTTACCAAAGCTGGGTACA‑CTGTCTATTACCCTATGCTGACTCAGAGTAAAGAGGACTTGGtctgtctc                                                                                                                                                                                                             >  1:407603/1‑294 (MQ=255)`  
                                                                                                  `ggTACATGATGAAATCCAAGTAGGCTGCCGTACCGAAGAGATTGCTCAGGTGGTCATTGAGACCGCACAAGAAGCGATGCGCTGGGTTGGAGACCACTGGAACTTCCGGTGTCTTCTGGATACCGAAGGTAAGATGGGTCCTAATTGGGCGATTTGCCACTGATACAGGAGGCTACTCATGAACGAAAGACACTTAACAGGTGCTGCTTCTGAAATGCTAGTAGCCTACAAATTTACCAAAGCTGGGTACA‑CTGTCTATTACCCTATGCTGACTCAGAGTAAAGAGGACTTGGTtgtatgt                                                                                                                                                                                                             >  1:662908/1‑301 (MQ=255)`  
                                                                                                  `ggTACATGATGAAATCCAAGTAGGCTGCCGTAACGAAGAGATTGCTCAGGTGGTCATTGAGACCGCACAAGAAGCGATGCGCTGGGTTGGAGACCACTGGAACTTCCGGTGTCTTCTGGATACCGAAGGTAAGATGGGTCCTAATTGGGCGATTTGCCACTGATACAGGAGGCTACTCATGAACGAAAGACACTTAACAGGTGCTGCTTCTGAAATGCTAGTAGCCTACAAATTTACCAAAGCTGGGTACA‑CTGTCTATTACCCTATGCTGACTCAGAGTAAAGAGGACTTGGTtgtatgt                                                                                                                                                                                                             <  1:553482/301‑1 (MQ=255)`  
                                                                                                  `atgtgtataagagacagaagtaggctgccgtaccgaAGAGATTGCTCAGGTGGTCATTGAGACCGCACAAGAAGCGATGCGCTTGGTTGGAGACCACTGGAACTTCCGGTGTCTTCTGGATACCGAAGGTAAGATGGGTCCTAATTGGGCGATTTGCCACTGATACAGGAGGCTACTCATGAACGAAAGACACTTAACAGGTGCTGCTTCTGAAATGCTAGTAGCCTACAAATTTACCAAAGCTGGGTACA‑CTGTCTATTACCCTATGCTGACTCAGAGTAAAGAGGACTTGGTTGTATGg                                                                                                                                                                                                             <  1:634674‑M1/284‑2 (MQ=255)`  
                                                                                                  `agagacagatgaaatccaaGTAGGCTGCCGTACCGAAGAGATTGCTCAGGTGGTCATTGAGACCGCACAAGAAGCGATGCGCTGGGTTGGAGACCACTGGAACTTCCGGTGTCTTCTGAATACCGAAGGTAAGATGGGTCCTAATTGGGCGATTTGCCACTGATACAGGAGGCTACTCATGAACGAAAGACACTTAACAGGTGCTGCTTCTGAAATGCTAGTAGCCTACAAATTTACCAAAGCTGGGTACA‑CTGTCTATTACCCTATGCTGACTCAGAGTAAAGAGGACTTGGTTGTATGg                                                                                                                                                                                                             <  1:629213‑M1/294‑2 (MQ=255)`  
                                                                                                      `cATGATGAAATCCAAGTAGGCTGCCGTACCGAAGAGATTGCTCAGGTGGTCATTGAGACCGCACAAGAAGCGATGCGCTGGGTTGGGGACCACTGGAACTTCCGGTGTCTTCTGGATACCGAAGGTAAGATGGGTCCTAATTGGGCGATTTGCCACTGATACAGGAGGCTACTCATGAACGAAAGACACTTAACAGGTGCTGCTTCTGAAATGCTAGTAGCCTACAAATTTACCAAAGCTGGGTACA‑CTGTCTATTACCCTATGCTGACTCAGAGTAAAGAGGACTTGGTTGTATGTAAgg                                                                                                                                                                                                         <  1:168118/301‑1 (MQ=255)`  
                                                                                                       `atgatgAAATCCAAGTAGGCTGCCGTACCGAAGAGATTGCTCAGGTGGTCATTGAGACCGCACAAGAAGCGATGCGCTGGGTTGGAGACCACTGGAACTTCCGGTGTCTTCTGGATACCGAAGGTAAGATGGGTCCTAATTGGGCGATTTGCCACTGATACAGGAGGCTACTCATGAACGAAAGACACTTAACAGGTGCTGCTTCTGAAATGCTAGTAGCCTACAAATTTACCAAAGCTGGGTACA‑CTGTCTATTACCCTATGCTGACTCAGAGTAAAGAGGACTTGGTTGTATGTAAGGa                                                                                                                                                                                                        >  1:301999/1‑301 (MQ=255)`  
                                                                                                       `atgatgAAAACCAAGTAGGCTGCCGTACCGAAGAGATTGCTCAGGTGGTCATTGAGACCGCACAAGAAGCGATGCGCTGGGTTGGAGACCACTGGAACTTCCGGTGTCTTCTGGATACCGAAGGTAAGATGGGTCCTAATTGGGCGATTTGCCACTGATACAGGAGGCTACTCATGAACGAAAGACACTTAACAGGTGCTGCTTCTGAAATGCTAGTAGCCTACAAATTTACCAAAGCTGGGTACA‑CTGTCTATTACCCTATGCTGACTCAGAGTAAAGAGGACTTGGTTGTATGTAAGGa                                                                                                                                                                                                        <  1:753123/301‑1 (MQ=255)`  
                                                                                                        `tgatgaAATCCAAGTAGGCTGCCGTACCGAAGAGATTGCTCAGGTGGTCATTGAGACCGCACAAGAAGCGATGCGCTGGGTTGGAGACCACTGGAACTTCCGGTGTCTTCTGGATACCGAAGGTAAGATGGGTCCTAATTGGGCGATTTGCCACTGATACAGGAGGCTACTCATGAACGAAAGACACTTAACAGGTGCTGCTTCTGAAATGCTAGTAGCCTACAAATTTACCAAAGCTGGGTACA‑CTGTCTATTACCCTATGCTGACTCAGAGTAAAGAGGACTTGGTTGTATGTAAGGac                                                                                                                                                                                                       >  1:480901/1‑300 (MQ=255)`  
                                                                                                          `gagacagccAAGTAGGCTGCCGTACCGAAGAGATTGCTCAGGTGGTCATTGAGACCGCACAAGAAGCGATGCGCTGGGTTGGAGACCACTGGAACTTCCGGTGTCTTCTGGATACCGAAGGTAAGATGGGTCCTAATTGGGCGATTTGCCACTGATACAGGAGGCTACTCATGAACGAAAGACACTTAACAGGTGCTGCTTCTGAAATGCTAGTAGCCTACAAATTTACCAAAGCTGGGTACA‑ATGTCTATTACCCTATGCTGACTCAGAGTAAAGAGGACTTGGTTGTATGTAAGGATgg                                                                                                                                                                                                     <  1:387557/294‑1 (MQ=255)`  
                                                                                                          `atgaAATCCAAGTAGGCTGCCGTACCGAAGAGATTGCTCAGGTGGTCATTGAGACCGCACAAGAAGCGATGCGATGGGTTGGAGACCACTGGAACTTCCGGTGTCTTCTGGATACCGAAGGTAAGATGGGTCCTAATTGGGCGATTTGCCACTGATACAGGAGGCTACTCATGAACGAAAGACACTTAACAGGTGCTGCTTCTGAAATGCTAGTAGCCTACAAATTTACCAAAGCTGGGTACA‑CTGTCTATTACCCTATGCTGACTCAGAGTAAAGAGGACTTGGTTGTATGgctgtccct                                                                                                                                                                                                     >  1:744577‑M1/1‑292 (MQ=255)`  
                                                                                                              `aaTCCAAGTAGGCTGCCGTACCGAAGAGATTGCTCAGGTGGTCATTGAGACCGCACAAGAAGCGATGCGCTGGGTTGGAGACCACTGGAACTTCCGGTGTCTTCTGGATACCGAAGGTAAGATGGGTCCTAATTGGGCGATTTGCCACTGATACAGGAGGCTACTCATGAACGAAAGACACTTAACAGGTGCTGCTTCTGAAATGCTAGTAGCCTACAAATTTACCAAAGCTGGGTACA‑CTGTCTATTACCCTATGCTGACTCAGAGTAAAGAGGACTTGGTTGTATGgaaggattgctgt                                                                                                                                                                                                 >  1:455763‑M1/1‑288 (MQ=255)`  
                                                                                                                `tCCAAGTAGGCTGCCGTACCGAAGAGATTGCTCAGGTGGTCATTGAGACTGCACAAGAAGCGATGCGCTGGGTTGGAGACCACTGGAACTTCCGGTGTCTTCTGGATACCGAAGGTAAGATGGGTCCTAATTGGGCGATTTGCCACTGATACAGGAGGCTACTCATGAACGAAAGACACTTAACAGGTGCTGCTTCTGAAATGCTAGTAGCCTACAAATTTACCAAAGCTGGGTACA‑CTGTCTATTACCCTATGCTGACTCAGAGTAAAGAGGACTTGGTTGTATGgaaggattgatgaca                                                                                                                                                                                               >  1:414388‑M1/1‑286 (MQ=255)`  
                                                                                                                `tCCAAGTAGGCTGCCGTACCGAAGAGATTGCTCAGGTGGTCATTGAGACCGCACAAGAAGCGATGCGCTGGGTTGGAGACCACTGGAACTTCCGGTGTCTTCTGGATACCGAAGGTAAGATGGATCCTAATTGGGCGATTTGCCACTGATACAGGAGGCTACTCATGAACGAAAGACACTTAACAGGTGCTGCTTCTGAAATGCTAGTAGCCTACAAATTTACCAAAGCTGGGTACA‑CTGTCTATTACCCTATGCTGACTCAGAGTAAAGAGGACTTGGTTGTATGTAAGGactgtctctt                                                                                                                                                                                               >  1:154173/1‑292 (MQ=255)`  
                                                                                                                  `cAAGTAGGCTGCCGTACCGAAGAGATTGCTCAGGTGGTCATTGAGACCGCACAAGAAGCGATGCGCTGGGTTGGAGACCACTGGAACTTCCGGTGTCTTCTGGATACCGAAGGTAAGATGGGTCCTAATTGGGCGATTTGCCACTGATACAGGAGGCTACTCATGAACGAAAGAAACTTAACAGGTGCTGCTTCTGAAATGCTAGTAGCCTACAAATTTACCAAAGCTGGGTACA‑CTGTCTATTACCCTATGCTGACTCAGAGTAAAGAGGACTTGGTGGTATGgaaggattgatgacatt                                                                                                                                                                                             >  1:613941‑M1/1‑284 (MQ=255)`  
                                                                                                                     `gTAGGCTGCCGTACCGAAGAGATTGCTCAGGTGGTCATTGAGACCGCACAAGAAGCGATGCGCTGGGTTGGAGACCACTGGAACTTCCGGTGTCTTCTGGATACCGAAGGTAAGATGGGTCCTAATTGGGCGATTTGCCACTGATACAGGAGGCTACTCATGAACGAAAGACACTTAACAGGTGCTGCTTCTGAAATGCTAGTAGCCTACAAATTTACCAAAGCTGGGTACA‑CTGTCTATTACCCTATACTGACTCAGAGTAAAGAGGACTTGGTTGGATGTAAGGATGGTAAATTTAGTa                                                                                                                                                                                          >  1:233865/1‑301 (MQ=255)`  
                                                                                                                            `gCCGTACCGCAGAGATTTCTCCGGTGGTCATTGAGACCGCACAAGAAGCGATGCGCTGGGTTGGAGACCACTGGAACTTCCGGTGTCTTCTGGATACCGAAGGTAAGATGGGTCCTAATTGGGCGATTTGCCACTGATACAGGAGGCTACTCATGAACGAAAGACACTTAACAGGTGCTGCTTCTGAAATGCTAGTAGCCTACAAATTTACCAAAGCTGGGTACA‑CTGTCTATTACCCTATGCTGACTAAGAGTAAAGAGGACTTGGTTGTATGTAAGGATGGTAAATTTAGTAAGGTTCa                                                                                                                                                                                   >  1:215300/1‑301 (MQ=255)`  
                                                                                                                              `cGTACCGAAGAGATTGCTCAGGTGGTCATTGAGACCGCACAAAAAGCGATGCGCTGGGTTGGAGACCACTGGAACTTCCGGTGTCTTCTGGATACCGAAGGTAAGATGGGTCCTAATTGGGCGATTTGCCACTGATACAGGAGGCTACTCATGAACGAAAGACACTTAACAGGTGCTGCTTCTGAAATGCTAGTAGCCTACAAATTTACCAAAGCTGGGTACA‑CTGTCTATTACCCTATGCTGACTCAGAGTAAAGAGGACTTGGTTGTATGTAAGGATGGTAAATTTAGTAAGGTTCAgg                                                                                                                                                                                 <  1:208310/301‑1 (MQ=255)`  
                                                                                                                               `gTACCGAAGAGATTGCTCAGGTGGTCATTGAGACCGCACAAGAAGCGATGCGCTGGGTTGGAGACCACTGGAACTTCCGGTGTCTTCTGGATACCGAAGGTAAGATGGGTCCTAATTGGGCGATTTGCCACTGATACAGGAGGCTACTCATGAACGAAAGACACTTAACAGGTGCTGCTTCTGAAATGCTAGTAGCCTACAAATTTACCAAAGCTGGGTACA‑CTGTCTATTACCCTATGCTGACTCAGAGTAAAGAGGACTTGGTTGTATGgaaggattgatgacattcgtagtacgtaca                                                                                                                                                                                >  1:285325‑M1/1‑271 (MQ=255)`  
                                                                                                                                 `aCCGAAGAGATTGCTCAGGTGGTCATTGAGACCGCACAAGAAGCGATGCGCTGGGTTGGAGACCACTGGAACTTCCGGTGTCTTCTGGATACCGAAGGTAAGATGGGTCCTAATTGGGCGATTTGCCACTGATACAGGAGGCTACTCATGAACGAAAGACACTTAACAGGTGCTGCTTCTGAAATGCTAGTAGCCTACAAATTTACCAAAGCTGGGTACA‑CTGTCTATTACCCTATGCTGACTCAGAGTAAAGAGGACTTGGTTGTATGTAAGGATGGTAAATTTAGTAAGGTTCAGGtct                                                                                                                                                                              >  1:729345/1‑299 (MQ=255)`  
                                                                                                                                 `aCCGAAGAGATTGCTCAGGTGGTCATTGAGACCGCACAAGAAGCGATGCGCTGGGTTGGAGACCACTGGAACTTCCGGTGTCTTCTGGATACCGAAGGTAAGATGGGTCCTAATTGGGCGATTTGCCACTGATACAGGAGGCTACTCATGAACGAAAGACACTTAACAGGTGCTGCTTCTGAAATGCTAGTAGCCTACAAATTTACCAAAGCTGGGTACA‑CTGTCTATTACCCTATGCTGACTCAGAGTAAAGAGGACTTGGTTGTATGTAAGGATGGTAAATTTAGTAAGGTTCAGGTTa                                                                                                                                                                              >  1:278838/1‑301 (MQ=255)`  
                                                                                                                                 `aCCGAAGAGATTGCTCAGGTGGTCATTGAGACCGCACAAGAAGCGATGCGCTGGGTTGGAGACCACTGGAACTTCCGGTGTCTTCTGGATACCGAAGGTAAGATGGGTCCTAATTGGGCGATTTGCCACTGATACAGGAGGCTACTCATGAACGAAAGACACTTAACAGGTGCTGCTTCTGAAATGCTAGTAGCCTACAAATTTACCAAAGCTGGGTACA‑CTGTCTATTACCCTATGCTGACTCAGAGTAAAGAGGACTTGGTTGTATGTAAGGATGGTAAATTTAGTAAGGTTCAGGTTa                                                                                                                                                                              >  1:654944/1‑301 (MQ=255)`  
                                                                                                                                   `agagacagttgctcaggtGGACATTGAGACCGCACAAGAAGCGATGCGCTGGGTTGGAGACCACTGGAACTTCCGGTGTCTTCTGGATACCGAAGGTAAGATGGGTCCTAATTGGGCGATTTGCCACTGATACAGGAGGCTACTCATGAACGAAAGACACTTAACAGGTGCTGCTTCTGAAATGCTAGTAGCCTACAAATTTACCAAAGCTGGGTACA‑CTGTCTATTACCCTATGCTGACTCAGAGTAAAGAGGACTTGGTTGTATGgaaggattgatgacattcgtagtacgtacatcat                                                                                                                                                                            <  1:129840‑M1/293‑35 (MQ=255)`  
                                                                                                                                              `cTCAGGTGGTCATTGAGACCGCACAAGAAGCGATGCGCTGGGTTGGAGACCACTGGAACTTCCGGTGTCTTCTGGATTCCGAAGGTAAGATGGGTCCTAATTGGGCGATTTGCCACTGATACAGGAGGCTACTCATGAACGAAAGACACTTAACAGGTGCTGCTTCTGAAATGCTAGTAGCCTACAAATTTACCAAAGCTGGGTACA‑CTGTCTATTACCCTATGCTGACTCAGAGTAAAGAGGACTTGGTTGTATGgaaggattgatgacattcgtagtacgtacatcatttcgtgaggcc                                                                                                                                                                 >  1:708463‑M1/1‑256 (MQ=255)`  
                                                                                                                                                  `ggtggtCATTGAGACCGCACAAGAAGCGATGCGCTGGGTTGGAGACCACTGGAACTTCCGGTGTCTTCTGGATACCGAAGGTAAGATGGGTCCTAATTGGGCGATTTGCCACTGATACAGGAGGCTACTCATGAACGAAAGACACTTAACAGGTGCTGCTTCTGAAATGCTAGTAGCCTACAAAATTACCAAAGCTGGGTACA‑CTGTCTATTACCCTATGCTGACTCAGAGTAAAGAGGACTTGGTTGTATGTAAGGATGGTAAATTTAGTAAGGTTCAGGttctgtctcttatacacatc                                                                                                                                                             >  1:533499/1‑283 (MQ=255)`  
                                                                                                                                                  `ggtggtCATTGAGACCGCACAAGAAGCGATGCGCTGGGTTGGAGACCACTGGAACTTCCGGTGTCTTCTGGATACCGAAGGTAAGATGGGTCCTAATTGGGCGATTTGCCACTGATACAGGAGGCTACTCATGAACGAAAGACACTTAACAGGTGCTGCTTCTGAAATGCTAGTAACCTACAAATTTACCAAAGCTGGGTACA‑CTGTCTATTACCCTATGCTGACTCAGAGTAAAGAGGACTTGGTTGTATGgaaggattgatgacattcgtagtacgtacatcatttcgtgaggccatta                                                                                                                                                             >  1:778036‑M1/1‑252 (MQ=255)`  
                                                                                                                                                      `gtCATTGAGACCGCACAAGAAGCGATGCGCTGGGTTGGAGACCACTGGAACTTCCGGTGTCTTCTGGATACCGAAGGTAAGATGGGTCCTAATTGGGCGATTTGCCACTGATACAGGAGGCTACTCATGAACGAAAGACACTTAACAGGTGCTGCTTCTGAAATGCTAGTAGCCTACAAATTTACCAAAGCTGGGTACA‑CTGTCTATTACCCTATGCTGACTCAGAGTAAAGAGGACTTGGTTGTATGgaaggattgatgacattcgtagtacgtacatcatttcgtgaggccattaagga                                                                                                                                                         <  1:139039‑M1/301‑54 (MQ=255)`  
                                                                                                                                                                 `ataagagacagcgatgggctGGGTTGGAGACCAATGGAACTTCCGGTGTATTCTGGATACCGAAGGTAAGATGGGTCCTAATTGGGCGATTTGCCACTGATACAGGAGGCTACTCATGAACGAAAGACACTTAACAGGTGCTGCTTCTGAAATGCTAGTAGCCTACAAATTTACCAAAGCTGGGTACA‑CTGTCTATTACCCTATGCTGACTCAGAGTAAAGAGGACTTGGTTGTATGgaaggattgatgacattcgtagtacgtacatcatttcgtgaggccattaaggacatgcacgaag                                                                                                                                              <  1:761487‑M1/292‑65 (MQ=255)`  
                                                                                                                                                                   `cacaAGAAGCGATGCGCTGGGTTGGAGACCACTGGAACTTCCGGTGTCTTCTGGATACCGAAGGTAAGATGGGTCCTAATTGGGCGATTTGCCACTGATACAGGAGGCTACTCATGAACGAAAGACACTTAACAGGTGCTGCTTCTGAAATGCTAGTAGCCTACAAATTTACCAAAGCTGGGTACA‑CTGTCTATTACCCTATGCTGACTCAGAGTAAAGAGGACTTGGTTGTATGTAAGGATGGTAAATTTAGTAAGGTTCAGGTTAAAACAGCCACAACGGTTCAAACCAACACAGgaga                                                                                                                                            >  1:256574/1‑301 (MQ=255)`  
                                                                                                                                                                      `agagacagATGCGCTGGGTTGGAGACCACTGGAACTTCCGGTGTCTTCTGGATACCGAAGGTAAGATGGGTCCTAATTGGGCGATTTGCCACTGATACAGGAGGCTACTCATGAACGAAAGACACTTAACAGGTGCTGCTTCTGAAATGCTAGTAGCCTACAAATTTACCAAAGCTGGGTACA‑CTGTCAATTACCCTATGCTGACTCAGAGTAAAGAGGACTTGGTTGTATGTAAGGATGGTAAATTTAGTAAGGTTCAGGTTAAAACAGCCACAACGGTTCAAACCAACACAGGAGATGc                                                                                                                                         <  1:369140/294‑1 (MQ=255)`  
                                                                                                                                                                        `gaagCGATGCTCTGGGTTGGAGACCACTGGAACTTCCAGTGTCTTCTGGATACCGAAGGTAAGATGGGTCCTAATTGGGCGATTTGCCACGGATACAGGAGGCTACTCATGAACGAAAGACACTTAACAGGTGCTGCTTCTGAAATGCTAGTAGCCTACAAATTTACCAAAGCTGGGTACA‑CTGTCTATTACCCTATGCTGACTCAGAGTAAAGAGGACTTGGTTGTATGgaaggattgatgacattcgtagtacgtacatcatttcgtgaggccattaaggacatgcacgaagagtatgc                                                                                                                                       <  1:309128‑M1/301‑72 (MQ=255)`  
                                                                                                                                                                          `agCGATGCGCTGGGTTGGAGACCACTGGAACTTCCGGTGTCTTCTGGATACCGAAGGTAAGATGGGTCCTAATTGGGCGATTTGCCACTGATACAGGAGGCTACTCATGAACGAAAGACACTTAACAGGTGCTGCTTCTGAAATGCTAGTAGCCTACAAATTTACCAAAGCTGGGTACA‑CTGTCTATTACCCTATGCTGACTCAGAGTAAAGAGGACTTGGTTGTATGgaaggattgatgacattcgtagtacgtacatcatttcgtgaggccattaaggacatgcacgaagagtatgcag                                                                                                                                     <  1:605662‑M1/301‑74 (MQ=255)`  
                                                                                                                                                                                     `gggTTGGAGACCACTGGAACTTCCGGTGTCTTCTGGATACCGAAGGTAAGATGGGTCCTAATTGGGCGATTTGCCACTGATACAGGAGGCTACTCATGAACGAAAGACACTTAACAGGTGCTGCTTCTGAAATGCTAGTAGCCTACAAATTTACCAAAGCTGGGTACA‑CTGTCTATTACCCTATGCTGACTCAGAGTAAAGAGGACTTGGTTGTATGTAAGGATGGTAAATTTAGTAAGGTTCAGGTTAAAACAGCCACAACGGTTCAAACCAACACAGGAGATGCCAAGCAGGTTAGGCt                                                                                                                          <  1:592470/301‑1 (MQ=255)`  
                                                                                                                                                                                     `gggTTGGAGACCACTGGAACTTCCGGTGTCTTCTGGATACCGAAGGTAAGATGGGTCCTAATTGGGCGATTTGCCACTGATACAGGAGGCTACTCATGAACGAAAGACACTTAACAGGTGCTGCTTCTGAAATGCTAGTAGCCTACAAATTTACCAAAGCTGGGTACA‑CTGTCTATTACCCTATGCTGACTCAGAGTAAAGAGGACTTGGTTGTATGTAAGGATGGTAAATTTAGTAAGGTTCAGGTTAAAACAGCCACAACGGTTCAAACCAACACAGGAGATGCCAAGCAGGTTAGGCt                                                                                                                          <  1:620910/301‑1 (MQ=255)`  
                                                                                                                                                                                                            `ggagatgtgtataagagacagggTAAGATGGGTCCTAATTGGGCGATTTGCCACTGATACAGGAGGCTACTCATGAACGAAAGACACTTAACAGGTGCTGCTTCTGAAATGCTAGTAGCCTACAAATTTACCAAAGCTGGGTACA‑CTGTCTATTACCCTATGCCGACTCAGAGTAAAGAGGACTTGGTTGTATGTAAGGATGGTAAATTTAGTAAGGTTCAGGTTAAAACAGCCACAACGGTTCAAACCAACACAGGAGATGCCAAGCAGGTTAGGCTAGGTGGATGCGGTAGGTCCGAat                                                                                                   <  1:340862/280‑1 (MQ=255)`  
                                                                                                                                                                                                             `caggtCTTCTGGATACCGAAGGTAAGATGGGTCCTAATTGGGCGATTTGCCACTGATACAGGAGGCTACTCATGAACGAAAGACACTTAACAGGTGCTGCTTCTGAAATGCTAGTAGCCTACAAATTTACCAAAGCTGGGTACA‑CTGTCTATTACCCTATGCTGACTCAGAGTAAAGAGGACTTGGTTGTATGTAAGGATGGTAAATTTAGTAAGGTTCAGGTTAAAACAGCCACAACGGTTCAAACCAACACAGGAGATGCCAAGCAGGTTAGGATAGGTGGATGCGGTAGGTCCGAata                                                                                                  <  1:116020/298‑1 (MQ=255)`  
                                                                                                                                                                                                              `gaataagagacagaCCGAAGGTAAGATGGGTCCTAATTGGGCGATTTGCCACTGATACAGGAGGCTACTCATGAACGAAAGACACTTAACAGGTGCTGCTTCTGAAATGCTAGTAGCCTACAAATTTACCAAAGCTGGGTACA‑CTGTCTATTACCCTATGCTGACTCAGAGTAAAGAGGACTTGGTTGTATGTAAGGATGGTAAATTTAGTAAGGTTCAGGTTAAAACAGCCACAACGGTTCAAACCAACACAGGAGATGCCAAGCAGGTTAGGCTAGGTGGATGCGGTAGGTCCGAatat                                                                                                 <  1:421801/288‑1 (MQ=255)`  
                                                                                                                                                                                                                    `tctGGATACCGAAGGTAAGATGGGTCCTAATTGGGCGATTTGCCACTGATACAGGAGGCTACTCATGAACGAAAGACACTTAACAGGTGCTGCTTCTGAAATGCTAGTAGCCTACAAATTTACCAAAGCTGGGTACA‑CTGTCTATTACCCTATGCTGACTCAGAGTAAAGAGGACTTGGTTGTATGTAAGGATGGTAAATTTAGTAAGGTTCAGGTTAAAACAGCCACAACGGTTCAAACCAACACAGGAGATGCCAAGCAGGTTAGGCTAGGTGGATGCGGTAGGTCCGAATATAAGGAt                                                                                           >  1:394771/1‑301 (MQ=255)`  
                                                                                                                                                                                                                      `tGGATACCGAAGGTAAGATGGGTCCTAATTGGGCGATTTGCCACTGATACAGGAGGCTACTCATGAACGAAAGACACTTAACAGGTGCTGCTTCTGAAATGCTAGTAGCCTACAAATTTACCAAAGCTGGGTACA‑CTGTCTATTACCCTATGCTGACTCAGAGTAAAGAGGACTTGGTTGTATGgaaggattgatgacattcgtagtacgtacatcatttcgtgaggccattaaggacatgcacgaagagtatgcagataaggactctttcaaacaatctcctgcaacagtacgggaggtg                                                                                         <  1:31822‑M1/301‑118 (MQ=255)`  
                                                                                                                                                                                                                       `ggctACCGAAGGTAAGATGGGTCCTAATTGGGCGATTTGCCACTGATACAGGAGGCTACTCATGAACGAAAGACACTTAACAGGTGCTGCTTCTGAAATGCTAGTAGCCTACAAATTTACCAAAGCTGGGTACA‑CTGTCTATTACCCTATGCTGACTCAGAGTAAAGAGGACTTGGTTGTATGTAAGGATGGTAAATTTAGTAAGGTTCAGGTTAAAACAGCCACAACGGTTCAAACCAACACAGGAGATGCCAAGCAGGTTAGGCGAGGTGGATGCGGTAGTTCCGAATATAAGGATggg                                                                                        >  1:123493/4‑300 (MQ=255)`  
                                                                                                                                                                                                                                `aGGTAAGATGGGTCCTAATTGGGCGATTTGCCACTGATACAGGTGGCTACTCATGAACGAAAGACACTTAACAGGTGCTGCTTCTGAAATGCTAGTAGCCTACAAATTTACCAAAGCTGGGTACA‑CTGTCTATTACCCTATGCTGACTCAGAGTAAAGAGGACTTGGTTGTATGTAAGGATGGTAAATTTAGTAAGGTTCAGGTTAAAACAGCCACAACGGTTCAAACCAACACAGGAGATGCCAAGCAGGTTAGGCTAGGTGGATGCGGTAGGTCCGAATATACGGATGGAGACTTTGAc                                                                               >  1:181240/1‑301 (MQ=255)`  
                                                                                                                                                                                                                                `aGGTAAGATGGGTCCTAATTGGGCGATTTGCCACTGATACAGGAGGCTACTCATGAACGAAAGACACTTAACAGGTGCTGCTTCTGAAATGCTAGTAGCCTACAAATTTACCAAAGCTGGGTACA‑CTGTCTATTACCCTATGCTGACTCAGAGTAAAGAGGACTTGGTTGTATGgaaggattgatgacattcgtagtacgtacatcatttcgtgaggccattaaggacatgcacgaagagtatgcagataaggactctttcaaacaatctcctgcaacagtacgggaggtgttctgactgt                                                                               >  1:84150‑M1/1‑174 (MQ=255)`  
                                                                                                                                                                                                                                 `ggTAAGATGGGTCCTAATTGGGCGATTTGCCACTGATACAGGAGGCTACTCATGAACGAAAGACACTTAACAGGTGCTGCTTCTGAAATGCTAGTAGCCTACAAATTTACCAAAGCTGGGTACA‑CTGTCTATTACCCTATGCTGACTCAGAGTAAAGAGGACTTGGTTGTATGgaaggattgatgacattcgtagtacgtacatcatttcgtgaggccattaaggacatgcacgaagagtatgcagataaggactctttcaaacaatctcctgcaacagtacgggaggtgttctgatgtct                                                                              >  1:578849‑M1/1‑173 (MQ=255)`  
                                                                                                                                                                                                                                 `ggTAAGATGGGTCCTAATTGGGCGATTTGCCACTGATACAGGAGGCTACTCATGAACGAAAGACACTTAACAGGTGCTGCTTCTGAAATGCTAGTAGCCTACAAATTTACCAAAGCTGGGTACA‑CTGTCTATTACCCTAGGCTGACTCAGAGTAAAGAGGACTTGGTTGTATGgaaggattgatgacattcgtagtacgtacattatttcgtgaggccattaaggacatgcacgaagagtatgcagataaggactctttcaaacaatctcctgcaacagtacgggaggtgttctgatgtct                                                                              >  1:100085‑M1/1‑173 (MQ=255)`  
                                                                                                                                                                                                                                 `ggTAAGATGGGTCCTAATTGGGCGATTTGCCACTGATACAGGAGGCTACTCATGAACGAAAGACACTTAACAGGTGCTGCTTCTGAAATGCTAGTAGCCTACAAATTTACCAAAGATGGGTACA‑CTGTCTATTACCCTATGCTGACTCAGAGTAAAGAGGACTTGGTTGTATGTAAGGATGGTAAATTTAGTAAGGTTCAGGTTAAAACAGCCACAACGGTTCAAACCAACCCAGGAGATGCCAAGCAGGTTAGGCTAGGTGGATGCGGTAGGTcctgtctcttatacacatctccgagcc                                                                              >  1:429221/1‑276 (MQ=255)`  
                                                                                                                                                                                                                                 `ggTAAGATGGGTCCTAATTGGGCGATTTGCCACTGATACAGGAGGCTACTCATGAACGAAAGACACTTAACAGGTGCTGCTTCTGAAATGCTAGGAGCCTACAAATTTACCAAAGCTGGGTACA‑CTGTCTATTACCCTATGCTGACGCAGAGTAAAGAGGACGTGGTTGTATGTAAGGATGGAAAATTTAGTAAGGTGCAGGTTAAAACAGCAACAACGGTTCAAACCAACAAAGGAGATGCCAAGCAGGGTAGGCTAGGTGGATGCGGTAGGTCCGAATATAAGGTTGGAGACTTTGaaa                                                                              >  1:494792/1‑299 (MQ=255)`  
                                                                                                                                                                                                                                  `gTAAGATGGGTCCTAATTGGGCGATTTGCCACTGATACAGGAGGCTACTCATGAACGAAAGACACTTAACAGGTGCTGCTTCTGAAATGCTAGTAGCCTACAAATTTACCAAAGCTGGGTACA‑CTGTCTATTACCCTATGCTGACTCAGAGTAAAGAGGACTTGGTTGTATGgaaggattgatgacattcgtagtacgtacatcatttcgtgaggccattaaggacatgcacgaagagtatgcagataaggactctttcaaacaatctcctgcaacagtacgggaggtgttctgactgtct                                                                             >  1:700490‑M1/1‑172 (MQ=255)`  
                                                                                                                                                                                                                                          `ggTCCTAATTGGGCGATTTGCCACTGATACAGGAGGCTACTCATGAACGAAAGACACTTAACAGGTGCTGCTTCTGAAATGCTAGTAGCCTACAAATTTACCAAAGCTGGGTACA‑CTGTCTATTACCCTATGCTGACTCAGAGTAAAGAGGACTTGGTTGTATGgaaggattgatgacattcgtagtacgtacatcatttcgtgaggccattaaggacatgcacgaagagtatgcagataaggactctttcaaacaatctcctgcaacagtacgggaggtgttctgatgtctgactacctg                                                                     >  1:401801‑M1/1‑164 (MQ=255)`  
                                                                                                                                                                                                                                                        `taagagacagtgatacaggagGCTACTCATGAACGAAAGACACTTAACAGGTGCTGCTTCTGAAATGCTAGTAGCCTACAAATTTACCAAAGCTGGGTACA‑CTGTCTATTACCCTATGCTGACTCAGAGTAAAGAGGACTTGGTTGTATGgaaggattgatgacattcgtagtacgtacatcatttcgtgaggccattaaggacatgcacgaagagtatgcagataaggactctttcaaacaatctcctgcaacagtacgggaggtgttctgatgtctgactacctgaaagtgctgcaagc                                                       <  1:585932‑M1/291‑152 (MQ=255)`  
                                                                                                                                                                                                                                                                         `ggaggCTACTCATGAACGAAAGACACTTAACAGGTGCTGCTTCTGAAATGCTAGTAGCCTACAAATTTACCAAAGCTGGGTACA‑CTGTCTATTACCCTATGCTGACTCAGAGTAAAGAGGACTTGGTTGTATGgaaggattgatgacattcgtagtacgtacatcatttcgtgaggccattaaggacatgcacgaagagtatgcagataaggactctttcaaacaatctcctgcaacagtacgggaggtgttctgatgtctgactacctgaaagtgctgcaagcaatcaaaagttgcccta                                      >  1:242431‑M1/1‑133 (MQ=255)`  
                                                                                                                                                                                                                                                                            `ggCTACTCATGAACGAAAGACACTTAACAGGTGCTGCTTCTGAAATGCTAGTAGCCTACAAATTTACCAAAGCTGGGTACA‑CTGTCTATTACCCTATGCTGACTCAGAGTAAAGAGGACTTGGTTGTATGgaaggattgatgacattcgtagtacgtacatcatttcgtgaggccattaaggacatgcacgaagagtatgcagataaggactctttcaaacaatctcctgcaacagtacgggaggtgttctgatgtctgactacctgaaagtgctgcaagcaatcaaaagttgccctaaga                                   >  1:464084‑M1/1‑130 (MQ=255)`  
                                                                                                                                                                                                                                                                                `aCTCATGAACGAAAGACACTTAACAGGTGCTGCTTCTGAAATGCTAGTAGCCTACAAATTTACCAAAGCTGGGTACA‑CTGTCTATTACCCTATGCTGACTCAGAGTAAAGAGGACTTGGTTGTATGTAAGGATGGTAAATTTAGTAAGGTTCAGGTTAAAACAGCCACAACGGTTCAAACCAACACAGGAGATGCCAAGCAGGTTAGGCTAGGTGGATGCGGTAGGTCCGAATATAAGGATGGAGACTTTGACATTCTTGCGGTTGAGGTTGACGAAGATGTGCTTATTTTCACATGGGAc                               >  1:807946/1‑301 (MQ=255)`  
                                                                                                                                                                                                                                                                                 `cTCATGAACGAAAGACACTTAACAGGTGCTGCTTCTGAAATGCTAGTAGCCTACAAATTTACCAAAGCTGGGTACA‑CTGTCTATTACCCTATGCTGACTCAGAGTAAAGAGGACTTGGTTGTATGTAAGGATGGTAAATTTAGTAAGGTTCAGGTTAAAACAGCCACAACGGTTCAAACCAACACAGGAGATGCCAAGCAGGTTAGGCTAGGTGGATGCGGTAGGTCCGAATATAAGGATGGAGACTTTGACATTCTTGCGGTTGTGGTTGACGAAGATGTGCTTATTTTCACATGGGACg                              >  1:517777/1‑301 (MQ=255)`  
                                                                                                                                                                                                                                                                                     `aagagacaggACACTTAACAGGTGCTGCTTCTGAAATGCTAGTAGCCTACAAATTTACCAAAGCTGGGTACA‑CTGTCTATTACCCTATGCTGACTCAGAGTAAAGAGGACTTGGTTGTATGTAAGGATGGTAAATTTAGTAAGGTTCAGGTTAAAACAGCCACAACGGTTCAAACCAACACAGGAGATGCCAAGCAGGTTAGGCTAGGTGGATGCGGAAGGTCCGAATATAAGGATGGAGACTTTGACATTCTTGCGGTTGTGGTTGACGAAGAAGTGCTTATTTTCACATGGGACGAAGt                          <  1:33330/292‑1 (MQ=255)`  
                                                                                                                                                                                                                                                                                           `aaaGACACTTAACAGGTGCTGCTTCTGAAATGCTAGTAGCCTACAAATTTACCAAAGCTGGGTACA‑CTGTCTATTACCCTATGCTGACTCAGAGTAAAGAGGACTTGGTTGTATGTAAGGATGGTAAATTTAGTAAGGTTCAGGTTAAAACAGCCACAACGGTTCAAACCAACACAGGAGATGCCAAGCAGGTTAGGCTAGGTGGATGCGGTAGGTCCGAATATAAGGATGGAGACTTTGACATTCTTGCGGTTGTGGTTGACGAAGATGTGCTTATTTTCACATGGGACGAAGTAAAAgg                    <  1:525768/301‑1 (MQ=255)`  
                                                                                                                                                                                                                                                                                                  `cTTAACAGGTGCTGCTTCTGAAATGCTAGTAGCCTACAAATTTACCAAAGCTGGGTACA‑CTGTCTATTACCCTATGCTGACTCAGAGTAAAGAGGACTTGGTTGTATGTAAGGATGGTAAATTTAGTAAGGTTCAGGTTAAAACAGCCACAACGGTTCAAACCAACACAGGAGATGCCAAGCAGGTTAGGCTAGGTGGATGCGGTAGGTCCGAATATAAGGATGGAGACTTTGACATTCTTGCGGTTGTGGTTGACGAAGATGTGCTTATTTTCACATGGGACGAAGTAAAAgctgtctct             >  1:201290/1‑293 (MQ=255)`  
                                                                                                                                                                                                                                                                                                     `aaCAGGTGCTGCTTCTGAAATGCTAGTAGCCTACAAATTTACCAAAGCTGGGTACA‑CTGTCTATTACCCTATGCTGACTCAGAGTAAAGAGGACTTGGTTGTATGTAAGGATGGTAAATTTAGTAAGGTTCAGGTTAAAACAGCCACAACGGTTCAAACCAACACAGGAGATGCCAAGCAGGTTAGGCTAGGTGGATGCGGTAGGTCCGAATATAAGGATGGAGACTTTGACATTCTTGCGGTTGTGGTTGACGAAGATGTGCTTATTTTCACATGGGACGAAGTAAAAGGTAAGACATcc          >  1:793702/1‑301 (MQ=255)`  
                                                                                                                                                                                                                                                                                                        `aGGTGCTGCTTCTGAAATGCTAGTAGCCTACAAATTTACCAAAGCTGGGTACA‑CTGTCTATTACCCTATGCTGACTCAGAGTAAAGAGGACTTGGTTGTATGTAAGGATGGTAAATTTAGTAAGGTTCAGGTTAAAACAGCCACAACGGTTCAAACCAACACAGGAGATGCCAAGCAGGTTAGGCTAGGTGGATGCGGTAGGTCCGAATATAAGGATGGAGACTTTGACATTCTTGCGGTTGTGGTTGACGAAGATGTGCTTATTTTCACATGGGACGAAGTAAAAGGTAAGACATCCAtg       <  1:263944/301‑1 (MQ=255)`  
                                                                                                                                                                                                                                                                                                         `ggTGCTGCTTCTGAAATGCTAGTAGCCTACAAATTTACCAAAGCTGGGTACA‑CTGTCTATTACCCTATGCTGACTCAGAGTAAAGAGGACTTGGTTGTATGgaaggattgatgacattcgtagtacgtacatcatttcgtgaggccattaaggacatgcacgaagagtatgcagataaggactctttcaaacaatctcctgcaacagtacgggaggtgttctgatgtctgactacctgaaagtgctgcaagcaatcaaaagttgccctaagactttccagtccaactatgtacggaacaat      >  1:55991‑M1/1‑101 (MQ=255)`  
                                                                                                                                                                                                                                                                                                          `gTGCTGCTTCTGAAATGCTAGTAGCCTACAAATTTACCAAAGCTGGGTACA‑CTGTCTATTACCCTATGCTGACTCAGAGTAAAGAGGACTTGGTTGTATGTAAGGATGGTAAATTTAGTAAGGTTCAGGTTAAAACAGCCTCAACGGTTCAAACCAACACAGGAGATGCCAAGCAGGTTAGGCTAGGTGGATGCGGTAGGTCCGAATATAAGGATGGAGACTTTGACATTCTTGCGGTTGTGGTTGACGAAGATGTGCTTATTTTCACATGGGACGAAGTAAAAGGTAAGACATCCAtgtg     >  1:545563/1‑301 (MQ=255)`  
                                                                                                                                                                                                                                                                                                            `gctgctTCTGAAATGCTAGTAGCCTACAAATTTACCAAAGCTGGGTACA‑CTGTCTATTACCCTATGCTGACTCAGAGTAAAGAGGACTTGGTTGTATGTAAGGATGGTAAATTTAGTAAGGTTCAGGTTAAAACAGCCACAACGGTTCAAACCAACACAGGAGATGCCAAGCAGGTTAGGCTAGGTGGATGCGGTAGGTCCGAATATAAGGATGGAGACTTTGACATTCTTGCGGTTGTGGTTGACGAAGATGTGCTTATTTTCACATGGGACGAAGTAAAAGGTAAGACATCCAtgtgtg   <  1:770169/301‑1 (MQ=255)`  

                                                                                                                                                                                                                                                                                                             `|`                                                                                                                                                                                                                                                                                                               
`GCTCTCATCTGCAAACTGTGGATTATCAAGACCGAAGAGATGCTCGTAGAGAAAGGCTTGAAGCATGGCTGGGATGGGGACTTTGCGTACATGGCATGGGTACATGATGAAATCCAAGTAGGCTGCCGTACCGAAGAGATTGCTCAGGTGGTCATTGAGACCGCACAAGAAGCGATGCGCTGGGTTGGAGACCACTGGAACTTCCGGTGTCTTCTGGATACCGAAGGTAAGATGGGTCCTAATTGGGCGATTTGCCACTGATACAGGAGGCTACTCATGAACGAAAGACACTTAACAGGTGCTGCTTCTGAAATGCTAGTAGCCTACAAATTTACCAAAGCTGGGTACA‑CTGTCTATTACCCTATGCTGACTCAGAGTAAAGAGGACTTGGTTGTATGTAAGGATGGTAAATTTAGTAAGGTTCAGGTTAAAACAGCCACAACGGTTCAAACCAACACAGGAGATGCCAAGCAGGTTAGGCTAGGTGGATGCGGTAGGTCCGAATATAAGGATGGAGACTTTGACATTCTTGCGGTTGTGGTTGACGAAGATGTGCTTATTTTCACATGGGACGAAGTAAAAGGTAAGACATCCATGTGTGT  >  NC_001604.1/16207‑16808`

  

Alignment Legend

Aligned base mismatch/match (shaded by quality score): `ATCG/ATCG` `< 3 ≤` `ATCG/ATCG` `< 19 ≤` `ATCG/ATCG` `< 30 ≤` `ATCG/ATCG` `< 37 ≤` `ATCG/ATCG` `< 38 ≤` `ATCG/ATCG`

Unaligned base: `atcg`    Masked matching base: `atcg`    Alignment gap: `‑`    Deleted base: `‑`

Reads not counted as support for junction

read\_name Not counted due to insufficient overlap past the breakpoint.

read\_name Not counted due to not crossing MOB target site duplication.
